# A novel end-to-end method to predict RNA secondary structure profile based on bidirectional LSTM and residual neural network

**DOI:** 10.1186/s12859-021-04102-x

**Published:** 2021-03-31

**Authors:** Linyu Wang, Xiaodan Zhong, Shuo Wang, Hao Zhang, Yuanning Liu

**Affiliations:** 1 College of Computer Science and Technology, Jilin University, Changchun, China; 2 Department of Pediatric Oncology, The First Hospital of Jilin University, Changchun, China

**Keywords:** RNA secondary structure profile, Residual neural network, LSTM, RPRes

## Abstract

**Background:**

Studies have shown that RNA secondary structure, a planar structure formed by paired bases, plays diverse vital roles in fundamental life activities and complex diseases. RNA secondary structure profile can record whether each base is paired with others. Hence, accurate prediction of secondary structure profile can help to deduce the secondary structure and binding site of RNA. RNA secondary structure profile can be obtained through biological experiment and calculation methods. Of them, the biological experiment method involves two ways: chemical reagent and biological crystallization. The chemical reagent method can obtain a large number of prediction data, but its cost is high and always associated with high noise, making it difficult to get results of all bases on RNA due to the limited of sequencing coverage. By contrast, the biological crystallization method can lead to accurate results, yet heavy experimental work and high costs are required. On the other hand, the calculation method is CROSS, which comprises a three-layer fully connected neural network. However, CROSS can not completely learn the features of RNA secondary structure profile since its poor network structure, leading to its low performance.

**Results:**

In this paper, a novel end-to-end method, named as “RPRes, was proposed to predict RNA secondary structure profile based on Bidirectional LSTM and Residual Neural Network.

**Conclusions:**

RPRes utilizes data sets generated by multiple biological experiment methods as the training, validation, and test sets to predict profile, which can compatible with numerous prediction requirements. Compared with the biological experiment method, RPRes has reduced the costs and improved the prediction efficiency. Compared with the state-of-the-art calculation method CROSS, RPRes has significantly improved performance.

## Background

RNA plays various important roles in the fundamental cellular processes and complex diseases, by means of transcription, replication, protein synthesis and gene expression regulation [1–4]. It usually binds to diverse proteins to participate in cell activities [5–7], and its structure determines its interaction and function [8, 9]. RNA possesses a three-tier structure, including the primary structure, secondary structure, and tertiary structure [10]. Of them, the primary structure is the base sequence, while the secondary structure is the planar structure formed through its own folding within the sequence, and the tertiary structure is formed in space by the interaction of secondary structural elements. Predicting secondary structure is an important basis for identifying the tertiary structure and an important prerequisite for understanding the RNA mechanisms of various biological activities [11]. Unfortunately, it is challenging to predict the secondary structure of RNA with different lengths, and most of the existing calculation methods are limited by the length of RNA [12–14]. RNA secondary structure profile can record whether each base is paired with others. The accurate prediction of RNA secondary structure profile not only helps to deduce the RNA secondary structure without length restriction [15] but also helps to identify the binding site of RNA, thus promoting the functional research of RNA.

RNA secondary structure profile can be obtained through the biological experiment and calculation methods. Of them, the biological experiment approach involves two ways, namely, chemical reagent and biological crystallization. The chemical reagent method utilizes different types of probes to obtain the RNA secondary structure profile, which can be classified into several categories according to the different probe types. Parallel Analysis of RNA Structure (PARS) [16–18] employs the catalytic activity of two enzymes RNase V1 (able to cut double-stranded bases) and S1 (able to cut single-stranded bases) to distinguishes double and single stranded bases. Selective 2–Hydroxyl Acylation analysed by Primer Extension (SHAPE) adopts the highly reactive chemical probes such as 1M6, NMIA(SHAPE) [19, 20], and NAI-N3 (icSHAPE) [21, 22] to characterize the RNA profile. Dimethyl sulfate (DMS) [23] uses the small size probe (CH3O)2SO2 to characterize RNA Profile. Although, the chemical reagent method can obtain a large number of prediction data, its cost is high and always linked with high noise, making it difficult to get results of all bases on RNA due to the limited of sequencing coverage [24]. The biological crystallization method can extract RNA from cells for crystallization and then obtain RNA secondary structure profile by using nuclear magnetic resonance (NMR) or x-ray crystallography [25]. The biological crystallization method can obtain accurate results, but heavy experimental work and high costs are needed. In this regard, it is necessary to adopt the calculation method to predict RNA secondary structure profile. The existing calculation method is CROSS [26], which creates a three-layer fully connected neural network and uses data generated from the biological experiment method as the training, validation, and test sets. In CROSS, each target base (the base will be predicted into profile) is processed into an RNA sequence with 13 bases, including 6 in the front and 6 in the rear of the target base, respectively. Those bases are encoded by one-hot coding before they are input into the CROSS. Hence, each piece of data is encoded into a vector of 152. There are 52 neurons in the input layer, 20 in the hidden layer, and 2 in the output layer. Its relatively small network space makes it hard to attain the expected performance. Therefore, it is necessary to propose a new time- and labor-saving calculation method to predict RNA secondary structure profile.

## Results

In this paper, a novel end-to-end prediction method “RPRes” was proposed based on Bidirectional LSTM (Bi-LSTM) [27, 28] and Residual Neural Network(ResNet) [29, 30]. RPRes and CROSS had both similarities and differences. The similarities were that both RPRes and CROSS were based on neural network, and the multiple data sets created by the biological experiment method were adopted for training, validation, and test; in this way, the characters of multiple biological experiment data sets were taken into account. The differences were that, on the one hand, the network space of RPRes was larger than that of CROSS, so it extracted and learned more features; on the other hand, the input data of RPRes contained more context information so that each target base was more comprehensively learned. RPRes first extracted the features of all target base data transmitted into the same format output based on a Bi-LSTM layer, and then a ResNet was used to classify the output of Bi-LSTM. During the method training, each of the data set was randomly divided into three sets: training set (80%), validation set (10%), and test set (10%). The training set was used to train the model, the validation set was used to select the excellent model, and the test set was used to test the model performance. During the method test and comparison, the performance of RPRes was tested and compared by the test set of all the biological experiment data, and its generalization ability was tested and compared by the test sets of different types of biological experiment data.

### Learning results and presentation

**Fig. 1:**
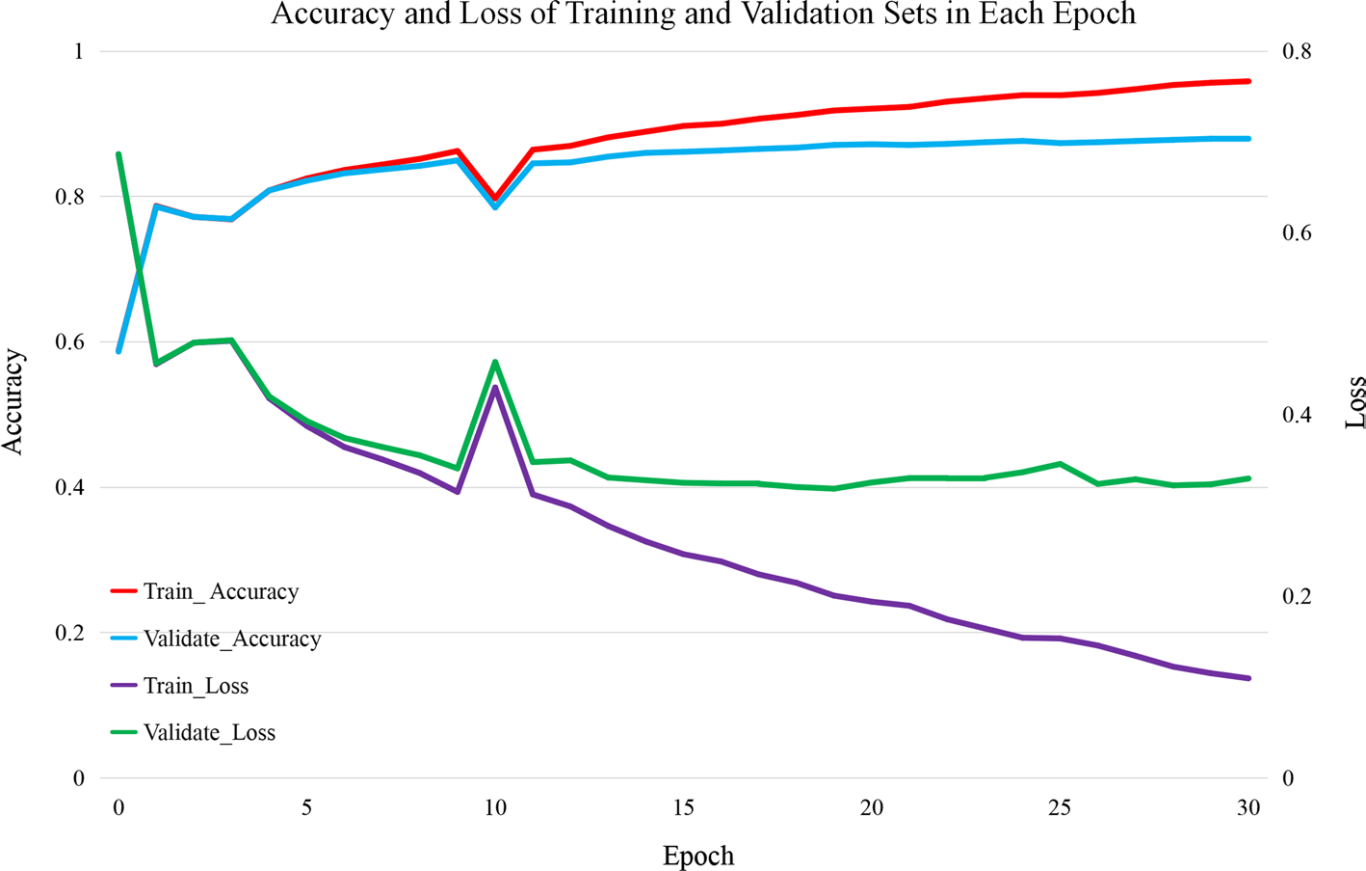
The accuracy and loss of training and validation sets, where the red and blue curves represent the accuracy of training and validation sets, while the violet and green curves represent the loss values of training and validation sets, respectively

In this section, the learning results of RPRes were presented. The data sets of human, mouse, zebrafish, PDB, and yeast were randomly divided into training (80%), validation (10%), and test (10%) sets. The merged training set(contains all training sets of multiple biological experiment data sets) and merged validation set(contains all validation sets of multiple biological experiment data sets) were used to training and validation RPRes for 30 epochs. Figure 1 presents the accuracy and loss of each epoch in the experiment. It can be seen that, although in the 10th epoch the curve fluctuated due to the sharp decline of the gradient, the overall accuracy of the merged validation set gradually increased and then stabilized, and the loss gradually decreased and then stabilized, indicating that RPRes can successfully predict the RNA secondary structure profile.

### Prediction results and comparison

**Fig. 2:**
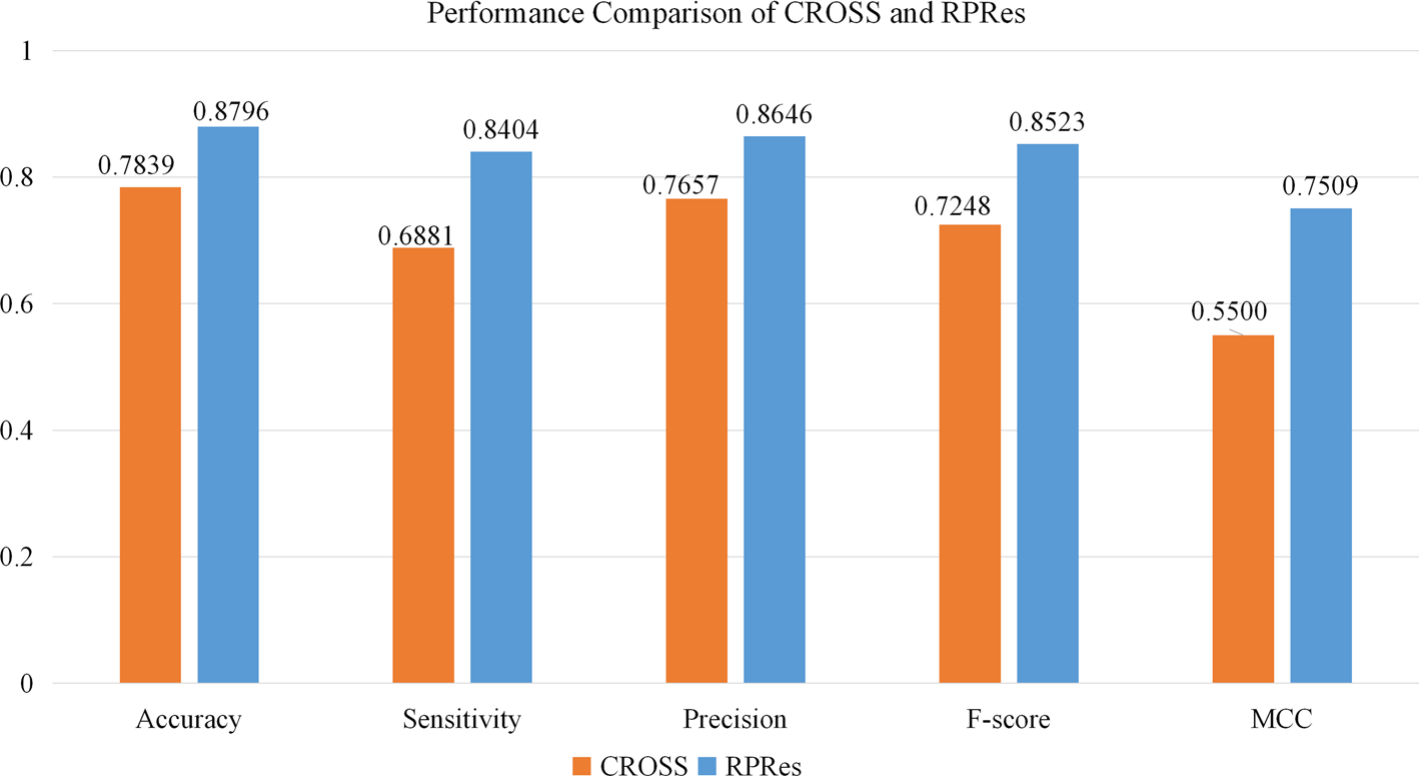
Comparison of performance between CROSS and RPRes. The blue column stands for the performance indexes of RPRes, while the orange column represents the performance indexes of CROSS

**Fig. 3:**
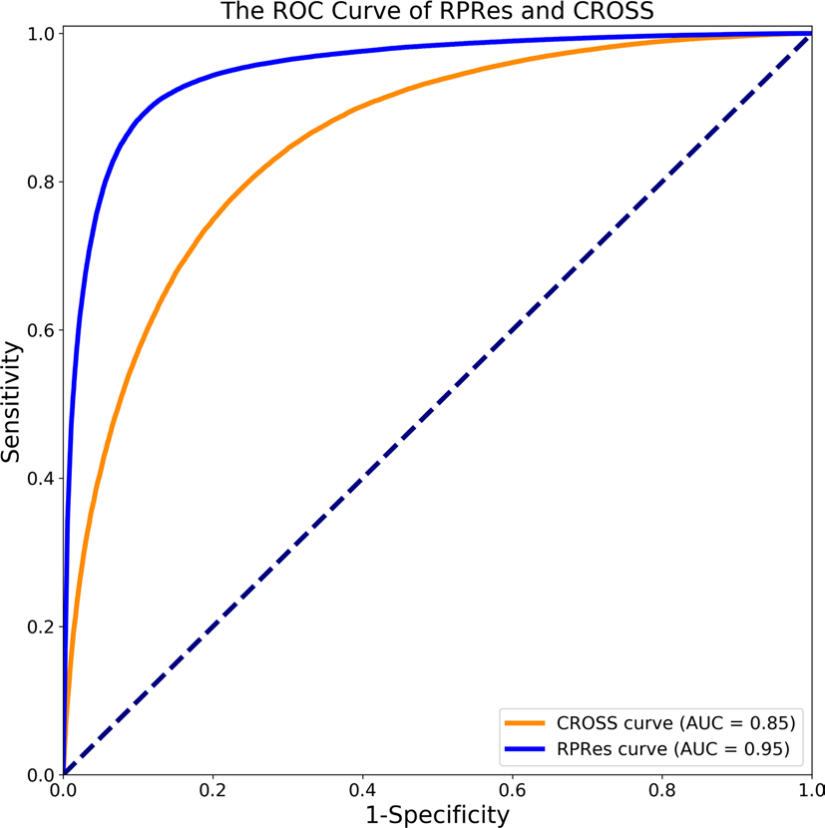
The ROC curves regarding the RPRes and CROSS predicted results, where the blue and orange curves represent RPRes and CROSS, respectively

In this section, the prediction results of RPRes were presented and compared with those of CROSS. To verify the performance of RPRes, we compared it with the state-of-the-art method CROSS from two aspects, namely, the performance in the merged test set(contains all test sets of multiple biological experiment data sets) and the generalization ability among the test sets of multiple biological experiment data sets. In the first aspect, the data sets of human, mouse, zebrafish, PDB, and yeast were randomly divided into training (80%), validation (10%) and test (10%) sets. The merged training, validation and test sets were adopted to training, validation and test the model, respectively. The indexes of Accuracy, Sensitivity, Precision, F-score, and MCC [31] were used to compare RPRes and CROSS, then the receiver operating characteristic(ROC) curves were plotted to present the predicted results of the two methods, and their area under the curve (AUC) values were compared. Accuracy was defined as the ratio of all the correctly predicted target bases to the total target bases, Sensitivity stood for the proportion of all the correctly predicted double-stranded bases to the total real double-stranded bases, Precision was the proportion of all the correctly predicted double-stranded bases to the total predicted double-stranded bases, F-score indicated the weighted harmonic mean of Sensitivity and Precision, while MCC was an index used to measure the classification performance of binary classification. Their corresponding formulas were shown below (Eqs. 1–5), where TP, TN, FP, and FN indicated True Positives, True Negative, False Positives, and False Negatives, respectively [32]. Figure 2 exhibits the comparison of performance between RPRes and CROSS. Obviously, RPRes achieved the best performance as far as all the indexes were concerned. Figure 3 shows the ROC curves of RPRes and CROSS. Clearly, the AUC value of RPRes was greater than that of CROSS.

1 $$\begin{aligned} Accuracy= \frac{TP + TN}{TP + TN + FP + FN} \end{aligned}$$

2 $$\begin{aligned} Sensitivity= \frac{TP}{TP + FN} \end{aligned}$$

3 $$\begin{aligned} Precision= \frac{TP}{TP + FP} \end{aligned}$$

4 $$\begin{aligned} F-score= \frac{2*TP}{2*TP + FP + FN} \end{aligned}$$

5 $$\begin{aligned} MCC= \frac{TP*TN-FP*FN}{\sqrt{(TP+FP)*(TP+FN)*(TN +FP)*(TN+FN)}} \end{aligned}$$

In the other aspect, the generalization ability of different methods was compared by cross-test among different test sets. To this end, the data sets of human, mouse, zebrafish, and PDB were randomly divided into training (80%), validation (10%), and test (10%) sets. Typically, all of the yeast data was created as a test set due to the small volume of yeast-related data. When one training set was applied to train the method, all of the validation sets were used to select the excellent model, and then all of the test sets were used to test the method. Tables 1 and 2 show the generalization accuracy of RPRes and CROSS. Clearly, the generalization accuracy of RPRes outperforms that of CROSS in most cases, except for the following datasets (human-yeast, mouse-PDB, and PDB-mouse) where the accuracy of CORSS was greater than that of RPRes.

**Table 1:** The generalization ability test of RPRes

Train	Test				
	Human	Mouse	Zebrafish	Yeast	PDB
Human	**0.9287**	**0.7866**	**0.6351**	0.7938	**0.5709**
Mouse	**0.7283**	**0.9452**	**0.6897**	**0.6895**	0.5847
Zebrafish	**0.6729**	**0.7291**	**0.8473**	**0.7443**	**0.5595**
PDB	**0.6526**	0.7539	**0.6234**	**0.5404**	**0.7952**

Bold values indicate the better generalization accuracy of RPRes

**Table 2:** The generalization ability test of CROSS

Train	Test				
	Human	Mouse	Zebrafish	Yeast	PDB
Human	0.8209	0.6608	0.6058	**0.8111**	0.5386
Mouse	0.6769	0.8870	0.6746	0.6829	**0.5942**
Zebrafish	0.6466	0.7017	0.8014	0.7108	0.5473
PDB	0.5784	**0.7723**	0.6135	0.5321	0.6588

Bold values indicate the better generalization accuracy of CROSS

## Discussion

**Fig. 4:**
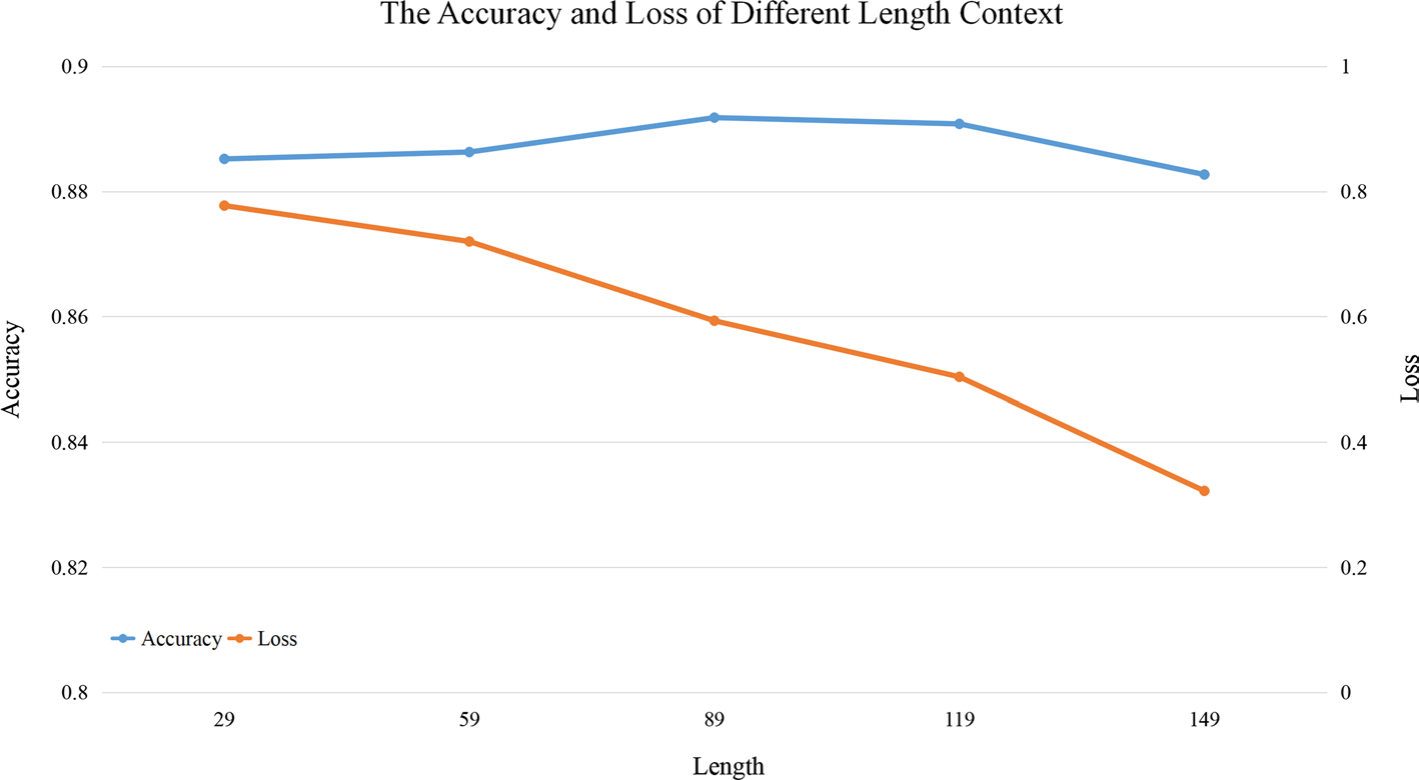
The accuracy and loss of RPRes in the context of various lengths. The blue curve represents the accuracy in different length context, while the orange curve represents the loss in different length context

RNA is a vital biological macromolecule involved in nearly all important life activities and complex diseases. The RNA profile records whether each base is paired with others, which facilitates to deduce its secondary structure and binding site. The traditional biological experiment methods for obtaining RNA profile is time-consuming and laborious, which can not meet the requirement of high-throughput data. In addition, the CROSS calculation method is a three-layer fully connected shallow neural network, whose relatively small network space may lead to its poor performance. Therefore, it is urgently needed to propose a new calculation method to accomplish profile prediction. In this paper, a novel end-to-end prediction method “RPRes” was proposed to predict RNA secondary structure profile based on Bi-LSTM and ResNet. Compared with the biological experiment method, RPRes only needed to input the data into the algorithm model to obtain the prediction results, which reduced the cost and improved the prediction efficiency. Compared with CROSS, RPRes, which had greatly improved network space and input data context, more efficiently extracted and learned the features of target base, thus improving its performance. To find the appropriate length of context, we intercepted different lengths of context sequence for target bases, including 149, 119, 89, 59, and 29. Figure 4 displays the accuracy and loss of different length contexts after training for 30 epochs. Clearly, the accuracy of different lengths context was close, but the loss value gradually decreased with the increase in length, suggesting the lower probability of overfitting and the higher generalization ability. Therefore, we cut 149 bases in the front and 149 bases in the rear of each target base, and used ‘N’ to pad the insufficient length sequences. Each target base was processed into the same format, as a result, the diversity of RNA sequence length did not affect RPRes.

In the learning process of RPRes, the accuracy of training and validation sets gradually increased, while the loss of both sets gradually decreased. In the last few epochs, the loss and accuracy of validation set tended to be stable, revealing that RPRes had stable performance and effectively predicted RNA secondary structure profile. In the process of method comparison, we compared RPRes and CROSS from two aspects. Firstly, we compared the performance of those two methods, trained and validated them using the same merged training and validation sets, and then compared the predicted results of the same merged test set. Specifically, their performances were measured by adopting five indexes: Accuracy, Sensitivity, Precision, F-score, and MCC and ROC curve. As observed from Figs. 3 and 4, RPRes increased the above five indexes by 12.20%, 22.13%, 12.91%, 17.59%, and 36.52%, respectively, and elevated the AUC value by 11.76%. Secondly, we compared the generalization ability of the two methods through the cross-test among different data sets. Tables 1 and 2 show the accuracy of RPRes and CROSS of different training sets. To conveniently display the specific improvements in the performance of each cross-test, Table 3 shows the performance improvement of RPRes in various cross-tests. Obviously, the performance of RPRes was improved in most cross-tests, and it was reduced only in human-yeast, mouse-PDB, and PDB-mouse. Such performance degradation was mainly observed in the cross-tests of two different types of data, namely, chemical reagent and biological crystallization. The main reason was that there were differences between the distribution of these two types of data, and the network space of CROSS was smaller, leading to the less learning of different characteristics, so the performance of CROSS was better than that of RPRes in those cross-tests. There are many aspects to evaluate the performance of the neural network model, among which, the two most important aspects are the performance of the same test set and the generalization ability of the cross-test set. RPRes was superior to CROSS in these two aspects, indicating that RPRes was effective and acceptable in predicting RNA profile. Two reasons might be mainly responsible for the superior performance of RPRes to CROSS. On the one hand, RPRes had a larger neural network space, which effectively learned and predicted the target bases. On the other hand, the training data (299 bases) were longer than those of CROSS (13 bases), which contained more features.

**Table 3:** The improvement of RPRes compared with CROSS in the generalization test

Train	Test				
	Human (%)	Mouse (%)	Zebrafish (%)	Yeast (%)	PDB (%)
Human	13.13	19.03	4.83	− 2.13	5.99
Mouse	7.59	6.56	2.23	0.96	− 1.59
Zebrafish	4.06	3.90	5.72	4.71	2.22
PDB	12.82	− 2.38	1.61	1.55	20.70

## Conclusions

RPRes is a novel prediction method based on deep learning. This method integrates various neural network technologies, and different technologies play different roles in its operation. For example, Bi-LSTM can extract the features of input data and integrate these data into outputs with the same format, making it convenient to identify them by the next layer network. ResNet classifies the output of Bi-LSTM into RNA profile. RPRes adopts diverse biological experimental data for learning and training, so it can learning the features of multiple different distribution data; as a result, the method is compatible with numerous prediction requirements. At the same time, compared with the biological experiment method, this method greatly reduces the prediction cost and its performance is improved compared with that of CROSS. Therefore, RPRes greatly contributes to predicting RNA secondary structure profile and studying RNA functions. Although RPRes is excellent in many aspects, it still has some defects. In future work, the performance and generalization ability of RPRes will be further improved. Meanwhile, the RNA profile prediction is basic research, which can be adopted to promote research on predicting RNA secondary structure and binding site. In future work, we will apply RNA profile prediction in biological experiments, so as to guide biologists to obtain more accurate results and contribute to life science research.

## Materials and method

### Data collection and processing

In this paper, multiple biological experiment data sets were selected as the training, validation, and test sets. Typically, the chemical reagent method data sets were obtained from two technologies, namely, PARS and icSHAPE, which covered the zebrafish [16], yeast [17], human [21], and mouse [21] genome-wide data. In addition, the biological crystallization method data set(PDB) was obtained from relevant literature [25], which contains 4667 RNA sequences. Since the original data can not be directly used as the input of RPRes, it is necessary to process those two types of data into the mature data.

The zebrafish and yeast data sets were obtained by the PARS technology. This technology scored each base in RNA according to the experimental results, where the positive values indicated double-stranded bases and the negative values indicated single-stranded bases. We sorted each base in RNA in line with the scores, and selected the five lowest values as the single-stranded target bases, whereas the five largest values as the double-stranded target bases. For zebrafish data, 53964 RNA sequences with 557425 target bases were screened, including 246853 double-stranded bases and 310572 single-stranded bases. For yeast data, 3196 RNA sequences with 34977 target bases were screened, including 18095 double-stranded bases and 16882 single-stranded bases.

The human and mouse data sets were obtained by the icSHAPE technology in chromatin, nucleoplasm, and cytoplasm, respectively, where some bases were scored both in vivo and vitro. Notably, the score of double-stranded base was close to 0, while that of single-stranded base was close to 1. During data screening, we screened target bases in chromatin, nucleoplasm, and cytoplasm, respectively. In human data, we selected at least three consecutive bases with available scores as the candidate bases. The double-stranded target base was defined as base with the scores of 0 both in vivo and vitro, while the single-stranded target base was base with the scores greater than 0.9 both in vivo and vitro. Those three target base sets were combined as the final human target bases after removing the duplicate data. In mouse data, we also selected at least three consecutive bases with available scores as the candidate bases. However, due to the inconsistent data among chromatin, nucleoplasm, and cytoplasm of mouse, there were certain differences in the parameters selected by those three data sets. In chromatin and nucleoplasm, double-stranded target base was base whose two consecutive base scores were 0 both in vivo and vitro, while single-stranded base was base whose two consecutive base scores were greater than 0.9 both in vivo and vitro. In nucleoplasm, double-stranded target base was defined as base with the scores of 0 both in vivo and vitro, while single-stranded base was base whose scores were greater than 0.9 both in vivo and vitro. These three target base sets were then integrated as the final mouse target bases after removing the duplicate data. For human data, 6221 RNA sequences with 399174 target bases were screened, including 263334 double-stranded bases and 135840 single-stranded bases. For mouse data, 11361 RNA sequences with 778032 target bases were screened, including 257969 double-stranded bases and 520063 single-stranded bases.

The original data obtained from the biological crystallization method contained a large number of unknown bases and redundant sequences. To eliminate the adverse effect on RPRes performance, RNA sequences containing unknown bases were removed and CD-HIT [33] was utilized to remove redundant sequences with identity greater than 80%. Finally, 502 RNA sequences were retained, which contained 225723 target bases, including 94750 double-stranded bases and 130973 single-stranded bases.

The profile of each target base was closely related to its context sequence. Based on the experimental results, we cut 149 bases in the front and 149 bases in the rear of each target base into a piece of data, and padded the insufficient length with ‘N’. In this way, each target base was made into a piece of data with the same length of 299 bases. Besides, these bases were encoded by one-hot coding, so that each piece of data was encoded as a 4299 matrix. Table 4 presents the rules of one-hot encoding.

**Table 4:** Bases and one-hot coding conversion rules

Bases	One-hot
A	1000
C	0001
G	0100
U	0010
N	0000

### Method

**Fig. 5:**
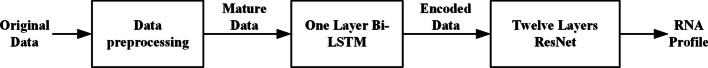
The Pipeline of the model consisting of three parts, including data preprocessing, Bi-LSTM, and ResNet. Those three parts cooperate with each other to predict RNA sequence as the RNA profile

RPRes was a comprehensive deep learning model that included Bi-LSTM [27, 28] and ResNet [29, 30]. RNA stands for the long-distance context-dependent sequential data, whose profile is closely related to the RNA context information. It is necessary to access the context features of each target base to predict its profile. LSTM is a special type of recurrent neural network, which can record the information of long-distance dependence of data. Hence, the Bi-LSTM was selected as the first layer in the model, extracted the context information of target base and its own information as the output with the same format, which facilitated the classification of the next layer network. ResNet is a modified deep convolution neural network, which combines the input data and the mapping data into the output data by using the shortcut connection, so that each layer of the network can contain real input data, which can effectively reduce the phenomenon of overfitting by the increase of layers. Hence, the ResNet was selected to classification of Bi-LSTM output. Figure 5 displays the pipeline of the model. During model running, when the original data were processed into mature data ($4*299$ matrixes), they were used as the model input. A layer of Bi-LSTM was utilized to encode the mature data into output with consistent format, and then a ResNet was used to classify the Bi-LSTM encoded data.

**Fig. 6:**
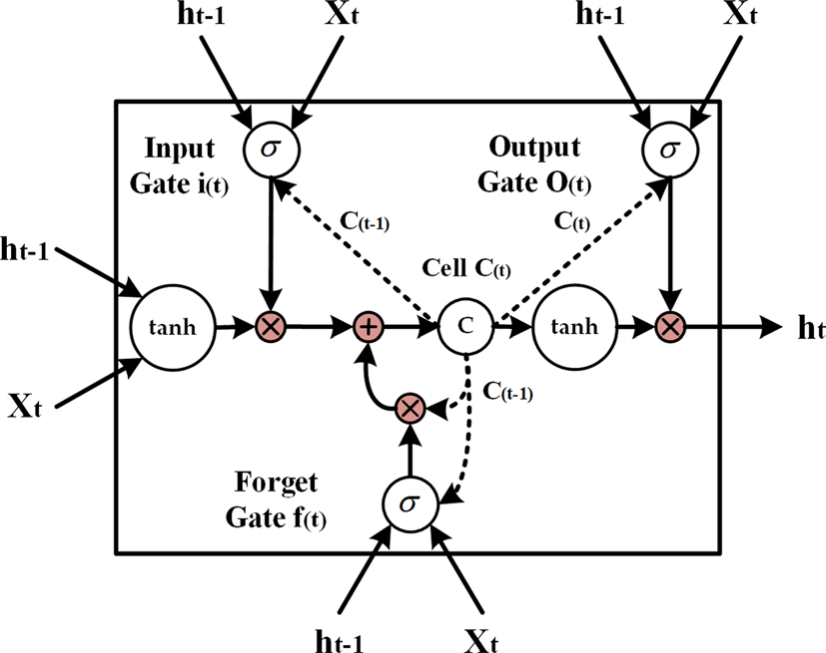
The diagram of LSTM cell, $i_{(t)}$, $f_{(t)}$, $o_{(t)}$, and $c_{(t)}$ are input gate, forget gate, output gate, and cell state, respectively

*Bi-LSTM:* Recurrent neural network (RNN) has been widely used in the research on text, audio, video, and other sequential data [34, 35]. When RNN is adopted to process sequential data, the output of neurons in a given moment serves as the input of neurons in the next moment; therefore, RNN can effectively utilize the context information. However, the traditional RNN has limited memory ability, which will lose its ability to learn information in the context with the increase in sequence length, making it easy to fall into gradient vanishing. LSTM, a special type of RNN, can solve the problem of gradient vanishing by introducing the gate mechanism; consequently, it outperforms the traditional RNN in representing context information and extracting long-distance dependency features from the sequential data. There are three gates in LSTM, including input gate, forget gate, and output gate. The input gate decides the new information to be stored in the cell state, the forget gate decides the information to be thrown away from the cell state, while the output gate decides the information to be used as output based on the cell state. Figure 6 is the diagram of LSTM cell, which can be implemented as follows (Eqs. 6–10):

6 $$\begin{aligned} i_t= \sigma (W_{xi} x_t + W_{hi}h_{t-1}+W_{ci}c_{t-1}+b_i) \end{aligned}$$

7 $$\begin{aligned} f_t= \sigma (W_{xf}x_t + W_{hf}h_{t-1}+W_{cf}c_{t-1}+b_f) \end{aligned}$$

8 $$\begin{aligned} c_t= f_t\odot c_{t-1}+i_t\odot tanh(W_{xc}x_t + W_{hc}h_{t-1} + b_c) \end{aligned}$$

9 $$\begin{aligned} o_t= \sigma (W_{xo}x_t + W_{ho}h_{t-1}+W_{co}c_{t}+b_o) \end{aligned}$$

10 $$\begin{aligned} h_t= o_t\odot tanh(c_{t}) \end{aligned}$$

where $\sigma$ represents the logistic sigmoid function, whereas i, f, o, and c stand for the input gate, forget gate, output gate, and cell vector, respectively, and all of these factors are at the same dimension as the hidden vector h. Meanwhile, w denotes the weight matrices while b indicates the bias vectors.

**Fig. 7:**
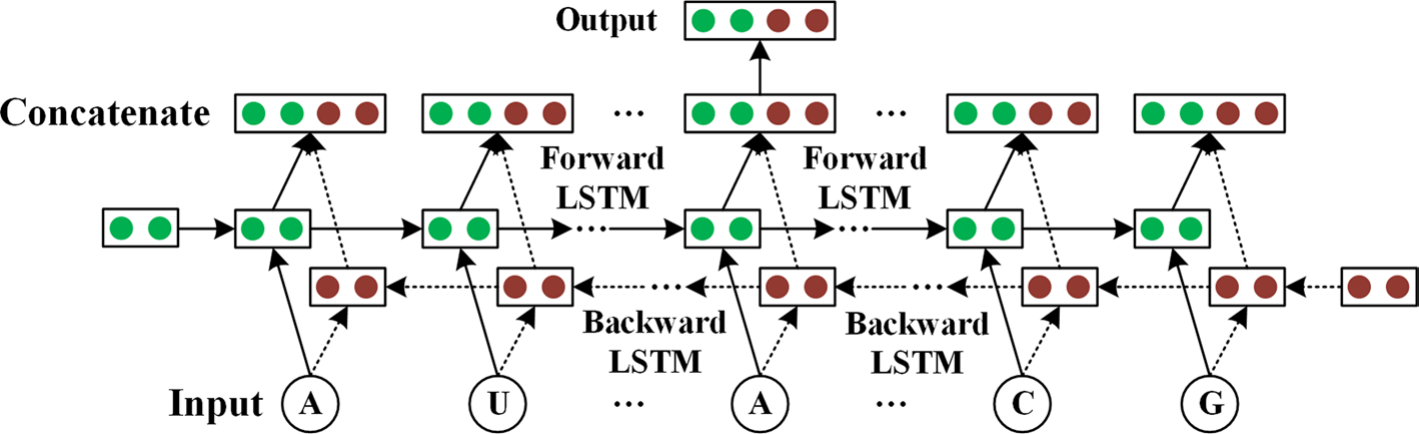
The diagram of Bi-LSTM. When RNA sequence is input into Bi-LSTM, two state vectors are obtained for each base. The concatenate vector of the target base is the output. The green and brown vectors are the states of Forward LSTM and Backward LSTM, respectively, where the dimension is $1*512$

In this paper, a one-layer Bi-LSTM was utilized to extract the context features of all target base data, which consisted of the forward and backward networks. The forward LSTM (512 hidden nodes) processed target sequence from left to right, whereas the backward LSTM (512 hidden nodes) processed target sequence in the reverse order. Therefore, two hidden state sequences were obtained, including one from the forward network and the other one from the backward network. Moreover, Bi-LSTM concatenated the forward and backward hidden states of each base, and the concatenate state (1024 nodes) of target base was the output. Figure 7 shows the diagram of Bi-LSTM.

**Fig. 8:**
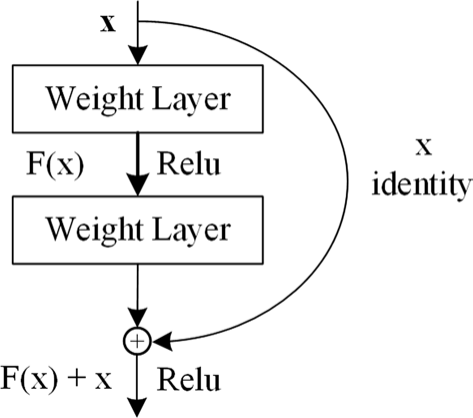
The schematic diagram of residual block. *x* is the input, *F*(*x*) stands for the mapping of residual block, *x* identity is the shortcut connection, and $F(x) + x$ represents the output of the residual block

*ResNet:* Deep convolutional neural networks [36] have been extensively utilized in the recognition and classification of two-dimensional data. It has been proved that the depth of deep convolutional neural network is of crucial importance, which helps to enrich the features and improve the accuracy [37]. However, the accuracy of convolutional neural network does not always increase with the increase of depth; instead, it may decline when the accuracy reaches saturation. When the convolutional neural network reaches saturation, to maintain its saturation accuracy if a new layer is added, the new layer must be an identity mapping layer: $H (x) = x$, which will result in the degeneration of deep network into a shallow network. Unfortunately, with the increase in layers, gradient vanishing or explosion may be encountered [38], making it difficult to fit the new layer into identity mapping. Hence, improving the depth of neural network alone can not meet the requirement of research. ResNet solves this problem effectively by means of the shortcut connection between the input conv layer and the output conv layer of each residual block [29]. Generally, ResNet contains a certain number of residual blocks, which are the core components of the ResNet model. Figure 8 [29] shows the schematic diagram of the residual block. In the residual block, the input is denoted as *x*, the mapping of the residual block is *F*(*x*), and the output $H(x) = F(x) + x$. In ResNet, when the network reaches saturation, *F*(*x*) is learning equal to 0, so the residual block becomes an identity map, which is much easier than the learning $H(x) = x$ in the ordinary convolution network [29]. In the process of backpropagation, the partial derivative of *H*(*x*) is shown in the following formula (Eq. 11).

11 $$\begin{aligned} \frac{\partial H}{\partial X} = \frac{\partial F}{\partial X} + 1 \end{aligned}$$

As observed, the result of partial derivative is close to 1. In this way, ResNet solves the problem of gradient vanishing and explosion, which reduces the training difficulty of neural network.

**Fig. 9:**
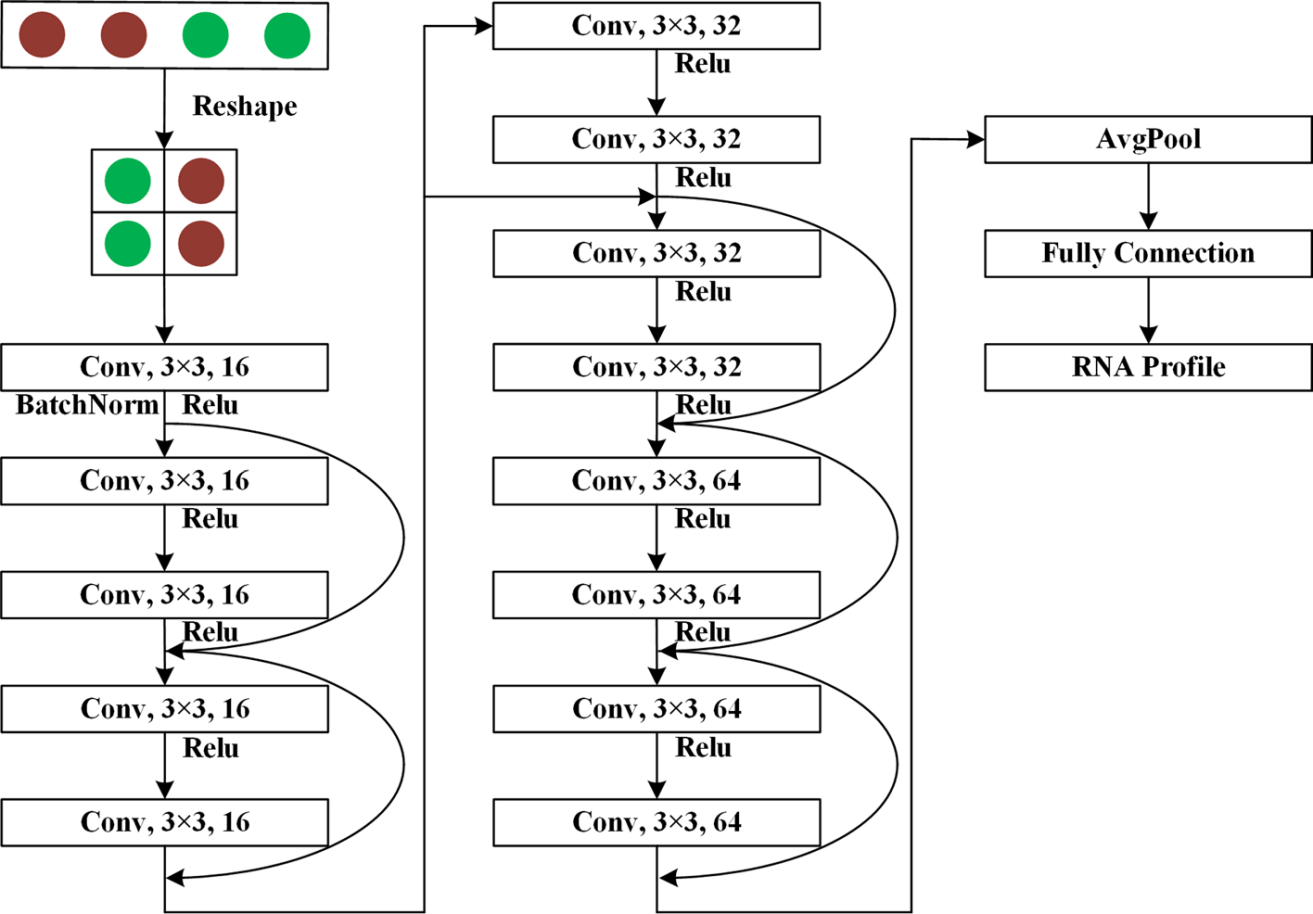
The schematic diagram of ResNet. Conv is the abbreviation of convolutional neural network, $3*3$ indicates the size of kernel, while 16, 32, and 64 are the dimensions of kernel

In this paper, ResNet involved three types of residual blocks, which had the same kernel of $3*3$, and the dimensions of the kernel were 16, 32, and 64, respectively. Figure 9 displays the schematic diagram of ResNet. It was observed from the figure that, the output ($1*1024$) of Bi-LSTM was first transformed into two-dimensional data ($32*32$) and then used as the input data of ResNet. In ResNet, a convolution neural network with 16-dimension $3*3$ convolution kernel was preferred to calculate the output data of Bi-LSTM, and the output contains 16-dimension $32*32$ data. Secondly, the residual blocks were used to calculate the output. The dimensions of convolution kernels of these residual blocks were 16, 16, 32, 32, 64, and 64, respectively. Therefore, the output of residual blocks was the 64-dimension $32*32$ data. Thereafter, the global average pooling layer was employed to pool the output data of the residual block into a $1*64$ vector. Finally, a fully connected layer was adopted to classify the output of residual block into the RNA secondary structure profile.

## Data Availability

All the original experimental data can be available from the citations, and the RPRes model can be available at https://github.com/linyuwangPHD/RPRes.
